# Transcription profile of *Trichophyton rubrum* conidia grown on keratin reveals the induction of an adhesin-like protein gene with a tandem repeat pattern

**DOI:** 10.1186/s12864-016-2567-8

**Published:** 2016-03-18

**Authors:** Tamires Aparecida Bitencourt, Claudia Macedo, Matheus Eloy Franco, Amanda Freire Assis, Tatiana Takahasi Komoto, Eliana Guedes Stehling, Rene Oliveira Beleboni, Iran Malavazi, Mozart Marins, Ana Lúcia Fachin

**Affiliations:** Unidade de Biotecnologia, Universidade de Ribeirão Preto, Av: Costábile Romano 2201, 14096-900 Ribeirão Preto, SP Brazil; Departamento de Genética, Faculdade de Medicina de Ribeirão Preto, Ribeirão Preto, Brazil; Faculdade de Ciências Farmacêuticas de Ribeirão Preto-USP, Ribeirão Preto, Brazil; Departamento de Genética e Evolução, Centro de Ciências Biológicas e da Saúde (CCBS), Universidade Federal de São Carlos, São Carlos, Brazil; Instituto Federal do Sul de Minas - Campus Machado, Machado, Brazil

**Keywords:** Adhesin-like protein, Conidia, Elastin, Keratin, Transcriptional gene expression, *Trichophyton rubrum*

## Abstract

**Background:**

*Trichophyton rubrum* is a cosmopolitan filamentous fungus that can infect human keratinized tissue (skin, nails and, rarely, hair) and is the major agent of all chronic and recurrent dermatophytoses. The dermatophyte infection process is initiated through the release of arthroconidial adhesin, which binds to the host stratum corneum. The conidia then germinate, and fungal hyphae invade keratinized skin structures through the secretion of proteases. Although arthroconidia play a central role in pathogenesis, little is known about the dormancy and germination of *T. rubrum* conidia and the initiation of infection*.* The objective of this study was to evaluate the transcriptional gene expression profile of *T. rubrum* conidia during growth on keratin- or elastin-containing medium, mimicking superficial and deep dermatophytosis, respectively*.*

**Results:**

A transcriptional profiling analysis was conducted using a custom oligonucleotide-based microarray by comparing *T. rubrum* conidia grown on elastin and keratin substrates. This comparison shows differences according to protein source used, but consisted of a very small set of genes, which could be attributed to the quiescent status of conidia. The modulated genes were related to the dormancy, survival and germination of conidia, including genes involved in the respiratory chain, signal transduction and lipid metabolism. However, an induction of a great number of proteases occurred when *T. rubrum* was grown in the presence of keratin such as the subtilisin family of proteases (Sub 1 and Sub 3) and leucine aminopeptidase (Lap 1 and Lap 2). Interestingly, keratin also promoted the up-regulation of a gene encoding an adhesin-like protein with a tandem repeat sequence. *In silico* analysis showed that the protein contains a domain related to adhesin that may play a role in host-pathogen interactions. The expression of this adhesin-like gene was also induced during the co-culture of *T. rubrum* with a human keratinocyte cell line, confirming its role in fungal-host interactions.

**Conclusion:**

These results contribute to the discovery of new targets involved in the adhesion of conidia and the maintenance of conidial dormancy, which are essential for triggering the process of infection and the chronicity of dermatophytosis.

## Background

*Trichophyton rubrum* is the main aetiological agent of human dermatophytoses, as well as all chronic and recurrent fungal infections in the world [1, 2]. Dermatophytes are adapted to infect keratinized tissues such as skin, hair and nails due to their ability to use keratin as a nutrient [3]. Although dermatophytes rarely penetrate beyond the epidermis, deeper penetration and systemic infections can occur in immunocompromised hosts [4]. Currently, *T. rubrum* has become an important public health problem due to an increase in invasive infections in immunocompromised patients [5, 6]. Analysis of the gene expression profile of fungi grown on culture medium containing protein substrates such as keratin and elastin, which mimic superficial and deep infections, respectively, can be used to understand fungal-host interactions [4, 7]. Additionally, the gene expression response of *T. rubrum* co-cultured on human keratinocytes can be evaluated.

The dermatophyte infection process is initiated through the release of arthroconidia adhesins, which bind to the host stratum corneum [8]. Most fungal adhesins contain an N-terminal carbohydrate or peptide-binding domain, central Ser- and Thr-rich domains, commonly in tandem repeats, and a C-terminal region that mediates covalent cross-linking to the wall through modified glycosylphosphatidylinositol (GPI) anchors [9]. Tandem repeats are adjacent DNA sequences 2–200 nucleotides in length. Some tandem repeats are involved in the pathogenicity of microorganisms and adaptation to a new environment [10]. Adhesins are considered the first line of a pathogen’s stratagem of host-cell invasion, and differences in adhesion have been associated with the greater pathogenicity/virulence of one strain over another [11]. Adhesins participate in mating, colony morphology changes, biofilm formation, fruiting body development, and interactions with mammalian and plant hosts. However, very few adhesins have been identified thus far in filamentous fungi [12].

After adhesion to the host’s skin, quiescent arthroconidia begin to germinate, leading to the formation of fungal hyphae that invade keratinized skin structures through the secretion of endo- and exoproteases [13]. Elucidation of this response of *T. rubrum* to the host may reveal new molecular targets that could be explored for the development of novel antifungal agents. These targets may be involved in the establishment and maintenance of fungal infection, and they may include genes that participate in the adhesion, dormancy and onset of the germination of conidia. The vast majority of studies on the gene expression of the fungal-host relationship in T. rubrum have used grown mycelium and then added skin fragments or protein substrates to the culture medium [14, 15]. However, arthroconidia are considered the primary infectious propagules that reach the skin and nails during infection in humans, and their germination is a crucial step in this process [16]. Therefore, the aim of the present study was to evaluated and compare the transcriptional profile of T. rubrum conidia during growth on keratin and elastin substrates by that contributing to the understanding of the infectious process of dermatophytes.

## Results

**Fig. 1:**
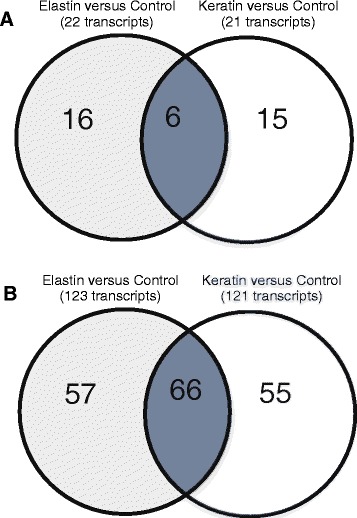
Venn diagrams showing the number of transcripts which are specifically up-regulated (**a**) and down-regulated (**b**) during the growth of *T. rubrum* on keratin or elastin compared to glucose minimal medium, respectively

The transcriptome profile of *T. rubrum* after growth on protein substrates was analysed using a microarray custom slide containing 6,091 sequences, which correspond to approximately 70 % of *T. rubrum* protein coding genes (according to the latest update released by the Broad Institute on 02/12/2014, available at www.broadinstitute.org/annotation/genome/dermatophyte_comparative). We identified 215 differentially expressed transcripts (P < 0.05, fold change ≥ 4) when the two growth conditions (keratin and elastin) were compared each one against the control (Cove’s minimal medium). The transcripts were mapped according to the Broad Institute database, and we found 145 and 142 transcripts that were modulated in the presence of elastin and keratin, respectively. Seventy-two differentially expressed transcripts were shared in both conditions (Fig. 1).

**Fig. 2:**
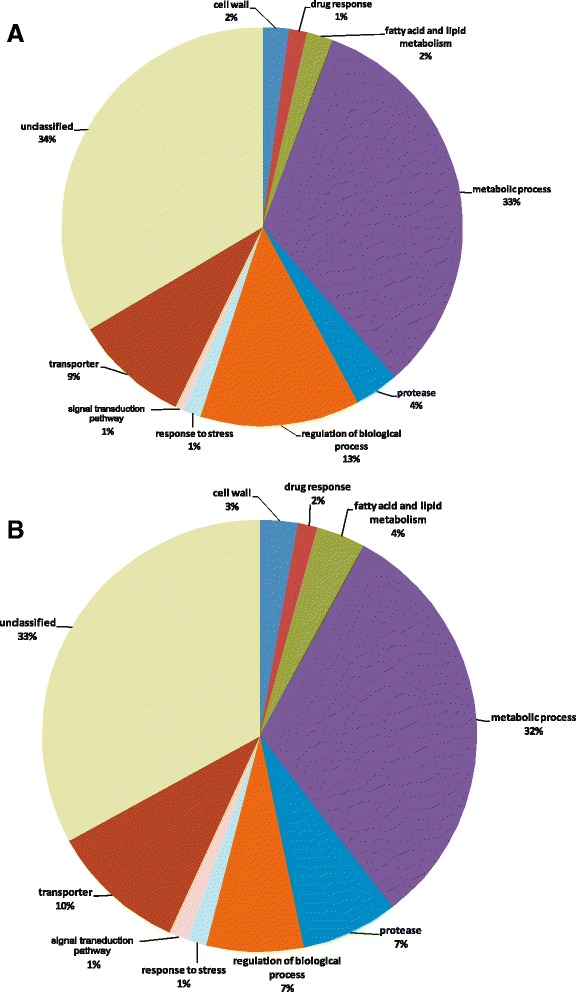
Functional annotation of genes modulated on keratin (**a**) and elastin (**b**) based on Gene Ontology

**Fig. 3:**
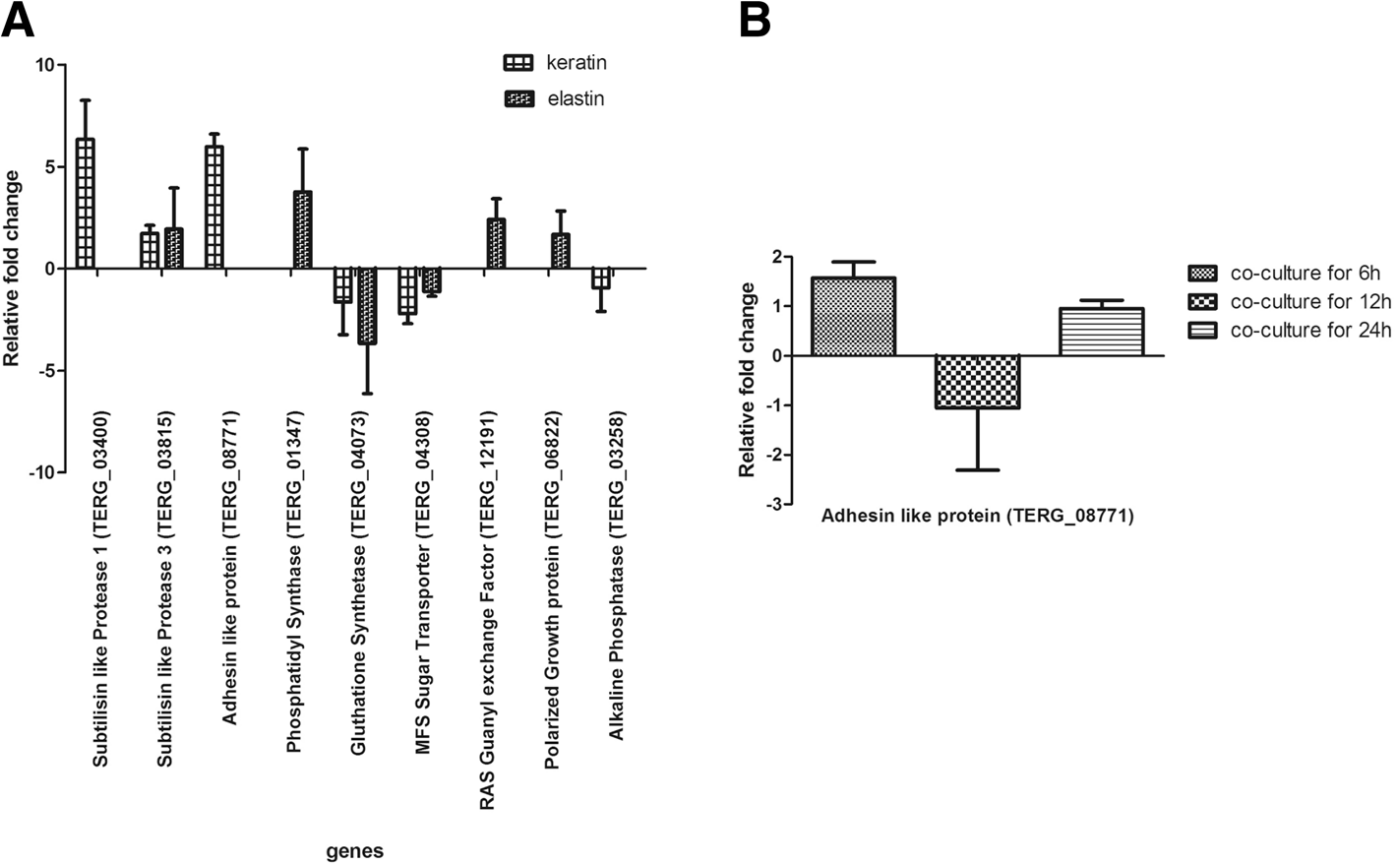
Real-time RT-PCR of selected genes from the microarray hybridization of *T. rubrum* genes during growth on keratin and elastin compared to control (**a**). Modulation of the adhesin-like protein gene of *T. rubrum* co-cultured with keratinocytes compared to fungal conidia (**b**)

Functional categorization of the genes differentially expressed on the two protein substrates identified genes involved in signal transduction, transport, drug resistance mechanisms, regulation of biological processes, response to stress, protease activity, fatty acid and lipid metabolism, the cell wall, and metabolic processes. Regarding to protease activity, the growth of *T. rubrum* conidia on keratin induced six protease genes that encoded respectively: leucine aminopeptidases Lap 1 and Lap 2; subtilisin-like proteins Sub 1, Sub3 and Sub 6; and metalloproteinases Mep3. Exclusive induction of the gene encoding Mep 4 was observed for conidia grown on keratin and elastin. The repression of 40 genes involved in metabolic processes was observed during the growth of *T. rubrum* conidia on protein substrates (keratin and elastin). On the other hand, genes related to the respiratory chain (NADP-dependent leukotriene b4 12-hydroxydehydrogenase) and tricarboxylic acid cycle (acyl enzyme) were induced. Besides genes differentially expressed in the two conditions, 30-34 % were found to be unclassified (Figs. 2a and b). Those genes exclusively modulated on keratin or elastin and those commonly modulated on both substrates are shown in Tables 1, 2 and 3, respectively. A subset of genes involved in different biological processes, such as adhesion (adhesin-like protein), dormancy (phosphatidyl synthase, polarized growth protein, Ras-guanyl exchange factor), protease secretion (subtilisins 1, 3 and alkaline phosphatase), and adaptation to nutritional stress (sugar MFS transporter and glutathione synthase), were validated by quantitative PCR (Fig. 3a), and shows a strong positive correlation.

**Table 1:** Genes exclusively modulated on keratin substrates

Gene ID	Tentative annotation	Expression change (n.fold)^a^
Metabolic process		
TERG_00499	Hypothetical protein	−4.88
TERG_02958	Assimilatory sulfite reductase	−6.51
TERG_03258	Alkaline phosphatase	−5.57
TERG_03339	Alternative oxidase	−4.69
TERG_03705	Cytochrome p450	−11.11
TERG_03706	Cytochrome p450	−5.68
TERG_05504	Thiol-specific antioxidant	−4.40
TERG_05628	GPI ethanolamine phosphate transferase 1	−5.17
TERG_06614	Formate dehydrogenase	−4.67
TERG_07017	NADP-dependent leukotriene b4 12-hydroxydehydrogenase	+4.47
TERG_07477	Copper-sulfate regulated protein 1	−4.17
TERG_07236	Hypothetical protein	−4.20
TERG_08353	Cytochrome p450	−8.40
TERG_07796	Short-chain dehydrogenase reductase family	−4.15
TERG_08140	2-Heptaprenyl- -naphthoquinone	−6.92
TERG_07777	O-acetylhomoserine –lyase	−4.58
TERG_12680	Alpha-mannosidase	−5.26
Regulation of biological process		
TERG_00222	CP2 transcription	+5.17
TERG_01360	C6 transcription factor	+4.79
TERG_08771	Adhesin like protein	+4.17
TERG_11518	Glucokinase	−4.25
TERG_07957	AP-1-like transcription factor	−6.07
TERG_08139	NAD dependent epimerase dehydratase family protein	−5.21
Transporter		
TERG_01994	OPT family oligopeptide transporter	−6.25
TERG_02616	Integral membrane protein	−6.15
TERG_03174	Siderochrome-iron transporter sit1	−5.95
TERG_03250	Monocarboxylate permease-like protein	−4.35
TERG_03928	Zinc-regulated transporter 1	−4.45
TERG_05199	MFS gliotoxin efflux transporter	−6.08
Protease activity		
TERG_02199	Glutamate carboxypeptidase	−4.70
TERG_02990	Subtilisin-like protease 6	+12.45
TERG_03248	Extracellular metalloproteinase 3	+5.81
TERG_03400	Subtilisin-like protease 1	+13.73
TERG_03815	Subtilisin-like protease 3	+9.66
TERG_05652	Leucine aminopeptidase 1	+6.62
TERG_08405	Leucine aminopeptidase 2	+10.74
Fatty acid and lipid metabolism		
TERG_01901	Glycerol kinase	−6.79
TERG_02984	Cytochrome p450 51	−5.13
TERG_05518	Short chain dehydrogenase	−5.35
TERG_11671	Phosphatidylserine synthase	−4.76
Cell wall		
TERG_04234	Hypothetical protein	−4.97
TERG_08178	Endoglucanase	+4.16
Signal transduction pathway		
TERG_04867	Sam and pH domain-containing protein	−5.17
TERG_05744	GTP-binding protein	−4.41
Drug response		
TERG_04952	Multidrug resistance protein	+4.78
Response to stress		
TERG_07058	HSP70 family	+4.47

^a^ Only genes with a fold change of four or higher are indicated. + induction; − repression

**Table 2:** Genes exclusively modulated on elastin substrates

Gene Id	Tentative annotation	Expression change (n.fold)^a^
Metabolic process		
TERG_00058	Oxidoreductase	−4.18
TERG_00563	GNAT family n-acetyltransferase	−5.67
TERG_00831	Gamma-glutamyltranspeptidase	−4.22
TERG_00852	Phytanoyl- dioxygenase	−4.57
TERG_01164	Beta-alanine synthase	−4.82
TERG_02340	Polysaccharide deacetylase	−4.85
TERG_02839	Nacht and ankyrin domain protein	−5.83
TERG_02842	6-Hydroxy-d-nicotine oxidase	−4.43
TERG_03695	Pyrroline-5-carboxylate reductase	+4.38
TERG_04310	Alcohol dehydrogenase	−4.62
TERG_04543	Classes i and ii family protein	−4.08
TERG_05299	Glutathione s-	−4.99
TERG_06147	Rhodocoxin reductase	−4.00
TERG_06160	Nitrite copper-containing	−4.69
TERG_06741	Ubiquitin c-terminal hydrolase	−4.54
TERG_07943	Hypothetical protein	−4.33
TERG_00830	Cytochrome p450	−4.05
TERG_01578	NB-ARC and ankyrin domain protein	−4.03
TERG_07083	Hypothetical protein	−4.04
Regulation of biological process		
TERG_00487	Hypothetical protein	−8.50
TERG_01003	37 s ribosomal protein rsm22	−4.90
TERG_01198	Pre-mRNA-splicing factor rse1	−5.29
TERG_02418	Translation initiation factor sui1	−31.53
TERG_05380	Protein kinase regulator ste50	−8.12
TERG_05655	An1 zinc finger protein	−4.46
TERG_05963	WD repeat protein	+4.40
TERG_06059	Helicase swr1	−7.58
TERG_06159	Hypothetical protein	−4.72
TERG_06729	Taz1-interacting factor 1	−4.49
TERG_06822	Polarized growth protein	+13.44
TERG_06891	C6 transcription factor	−5.52
TERG_08611	E3 Ubiquitin ligase complex scf subunit sconc	−4.33
Transporter		
TERG_01336	MFS transporter	−4.33
TERG_01353	V-type c subunit family protein	−9.94
TERG_02333	Acetyl-coenzyme A transporter 1	+4.23
TERG_02545	MFS monocarboxylate transporter	−4.89
TERG_02654	MFS amine transporter	−4.70
TERG_03907	Amino acid transporter	+5.60
TERG_04093	K+ homeostasis protein kha1	−4.21
TERG_12078	FMN-binding split barrel-like protein	−4.31
TERG_12574	Tmem1 family	−6.62
Protease activity		
TERG_02988	Asparaginase	−4.30
Fatty acid and lipid metabolism		
TERG_01347	Phosphatidyl synthase	+5.40
TERG_12530	3-Ketoacyl- thiolase	+4.07
Cell wall		
TERG_03843	Chitin synthase b	+4.25
Signal transduction pathway		
TERG_12191	Ras guanyl-nucleotide exchange factor	+4.33
TERG_07570	G-protein signaling- receptor signaling pathway	−4.11
Drug response		
TERG_01820	MFS drug transporter	−4.61
TERG_05575	MFS multidrug transporter	−4.01
Response to stress		
TERG_02795	Thiazole biosynthetic mitochondrial	−7.22

^a^ Only genes with a fold change of four or higher are indicated. + induction; −repression

**Table 3:** *T. rubrum* genes modulated on both keratin and elastin substrates

Gene Id	Expression change (n-fold)^a^		Tentative annotation
	Keratin vs control	Elastin vs control	
Metabolic Process			
TERG_00072	−6.70	−6.73	Hypothetical protein
TERG_00073	−5.98	−7.40	NADPH dehydrogenase
TERG_00374	−6.72	−5.09	Metallophosphoesterase domain-containing protein 2
TERG_00881	−5.18	−5.39	Reticulon-4-interacting protein 1
TERG_01338	−6.84	−5.30	Hydantoinase
TERG_02078	−7.57	−5.84	Thiamine biosynthesis protein
TERG_02132	−8.95	−10.39	5-Histidylcysteine sulfoxide synthase
TERG_02133	−4.79	−6.16	Flug protein
TERG_02134	−10.00	−10.03	Indoleamine -dioxygenase-like protein
TERG_02197	−4.85	−4.49	Aliphatic nitrilase
TERG_02217	−5.99	−4.87	GNAT family protein
TERG_02538	−4.95	−4.85	Carboxylesterase family protein
TERG_02712	−44.76	−43.66	Glutamyl-tRNA amidotransferase
TERG_03707	−12.95	−5.49	Fusicoccadiene synthase
TERG_04073	−4.98	−6.64	Glutathione synthetase
TERG_06261	−6.17	−5.18	Phosphoric ester hydrolase
TERG_07159	−4.91	−4.72	Prenyltransferase alpha subunit
TERG_07504	−5.71	−6.65	Carbohydrate-binding protein
TERG_07821	−8.24	−11.57	Hypothetical protein
TERG_08054	−6.35	−7.18	Homoserine acetyltransferase family protein
TERG_08261	−4.47	−5.79	Glutamate decarboxylase
TERG_08554	−4.37	−6.20	Riboflavin-specific deaminase
TERG_08787	−7.01	−5.61	Aminotransferase family protein
TERG_08868	−5.02	−4.38	FKBP-type peptidyl-prolyl
TERG_06540	−4,10	−7.32	Glutathione transferase
Regulation of Biological Process			
TERG_01762	−7.45	−4.30	Sulfite reductase beta-component
TERG_03972	−4.43	−5.42	Elongation factor g
TERG_04862	−11.93	−7.97	C6 sexual development transcription factor
TERG_05617	−6.98	−8.13	Hypothetical protein
TERG_08437	−8.34	−6.36	C2H2 transcription factor
TERG_11890	−8.78	−6.75	Hypothetical protein
Transporter			
TERG_04308	−20.09	−13.39	MFS sugar transporter
TERG_06954	−10.53	−4.10	Hypothetical protein
Protease Activity			
TERG_02001	−17.10	−14.96	Dipeptidyl-peptidase 5
TERG_03104	−7.94	−10.17	Signal peptidase i
TERG_04324	+14.97	+4.77	Extracellular metalloproteinase 4
TERG_05842	−4,08	−5.55	Peptidase
Fatty acid and lipid metabolism			
TERG_02704	−12.05	−19.34	Short-chain dehydrogenase
TERG_05484	+6.56	+5.30	Acyl- dehydrogenase
TERG_11720	−4.39	−4.65	Acyl- dehydrogenase
Cell Wall			
TERG_00060	−9.15	−7.25	Bys1 domain
TERG_05625	−15.77	−7.94	Glycoside hydrolase family 18 protein
TERG_11657	−6.60	−9.36	Glycoside hydrolase family 18 protein
Drug Response			
TERG_05309	−5.85	−6.39	Puromycin resistance protein pur8
Response to stress			
TERG_01122	+6.72	+6.75	Chaperone heat shock protein

^a^Only genes with a fold change of four or higher are indicated. + induction; −repression

Among those, one gene drew our attention, an adhesin-like protein upregulated in *T. rubrum* conidia grown in keratin substrate and containing an interesting pattern of tandem repeat sequences related to adhesion and virulence. This gene was also induced when *T. rubrum* conidia were co-cultured with a human keratinocyte cell line for 6 h and 24 h (Fig. 3b). Importantly, the induction of this gene was not observed in a microarray analysis using *T. rubrum* mycelium grown on the same protein substrate (data not shown), suggesting that this gene might play a role in the early stages of infection. The function of an adhesin-like protein was predicted using FaaPred software, with the gene showing a high confidence score (0.997).

**Fig. 4:**
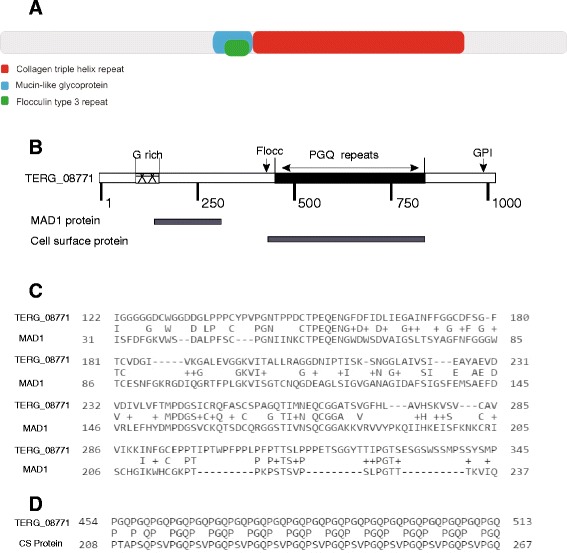
Structural features of TERG_08771 adhesin-like protein **a**) Main domains in the adhesin-like protein sequence according to the NCBI’s conserved domain database. **b** Schematic structure of TERG_08771 showing a glycine rich region, tandem repeats of proline, glycine and glutamine, and a glycosylphosphatidylinositol anchor site (GPI). **c** Alignment of the homologous region between TERG_08771 and MAD1. **d** Conservation of tandem repeat regions between TERG_08771 and cell surface protein (CS protein) of *Aspergillus fumigatus*

Besides the in *silico* characterization of this gene indicated a sequence of 3,030 bp (GenBank Database under the accession number: 327302703), containing a tandem repeat sequence. The tandem repeat pattern is a minisatellite type that shows high variability among dermatophyte species and strains and is located between positions 1,382 and 2,425, with a consensus region of 45 bp and a total length of 1,044 bp. The tandem repeat sequence encodes 348 amino acids; has a repeat unit of glycine, glutamine and proline; and is characterized by the presence of a collagen triple helix domain preceded by a mucin-like glycoprotein domain and flocculin type 3 domain (Fig. 4a). In addition the similarities between MAD1 (*Metarhizium anisopliae* adhesin) and the *T. rubrum* adhesin-like protein was verified and exist at the N-terminus, starting in the glycine-rich region. Furthermore, both proteins share a predicted GPI cell wall anchor site at the C-terminus and exhibit a tandem repeat sequence in the mid-region (Figs. 4b and c). Similarly, the findings showed similarities between the *T. rubrum* adhesin-like protein and a cell surface protein of *Aspergillus fumigatus* (*cspA* - Afu3g08990), which is characterized by a 188-amino acid serine/threonine/proline-rich N-terminus followed by a large, variable, six-amino acid serine/proline [PGQPS (A/V)] tandem repeat region (Fig. 4d). In the last case, besides the tandem repeats, both proteins also contain collagen and flocculin domains and a GPI anchor site.

**Fig. 5:**
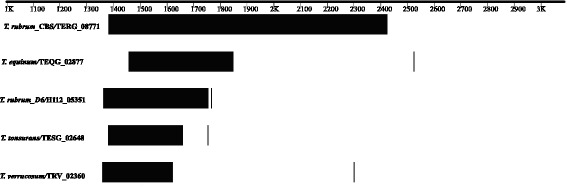
Variable numbers of tandem repeat sequences in the adhesion-like protein gene of different dermatophytes. The vertical bars represent the end of the transcribed genes of the different species and strains

The adhesin-like protein gene of *T. rubrum* has homologous genes in dermatophyte species, which indicate that the repetitive units are conserved between species. Variation of the extent of the tandem repeats can be observed among species and strains (Fig. 5).

## Discussion

The establishment of *T. rubrum* infection initiates by adhesion to the tissue surface mediated through the release of carbohydrate-binding adhesins by arthroconidia that bind to surface of host receptors [8, 17, 18]. The conidia in the dormant stage begins to germinate and then hyphae readily penetrate the stratum corneum, preventing the fungus to be disconnected from the skin due to flaking of the keratinized epithelium [13]. After adhesion, dermatophytes secrete a wide range of enzymes such as proteases, keratinases, lipase, elastase, collagenase, phosphatases and esterases, which are important factors during the infectious process [19–21]. The keratinase secreted by dermatophytes catalyze the degradation of keratin present in the host tissue into oligopeptides or amino acids, which can then be assimilated by the fungi [22].

In this study, the use of different protein sources such as keratin and elastin did not induce a profile of gene expression which would be characteristic of the superficial or deep infection, respectively. This could be attributed to the dormant stage of conidia, because the status of quiescence may be responsible for slow transcriptional profile due to starvation of nutrients. However, it is believed that there is a specific induction of proteases with respect to the protein source used [23]. In this work it was observed in fact that fungal growth in the presence of keratin promoted the induction of a greater number of proteases, specifically of the subtilisin family of proteases (Sub 1 and Sub 3) and leucine aminopeptidase (Lap 1 and Lap 2) compared to growth of the fungus in elastin.

### *In silico* identification and prediction of a gene coding an adhesin-like protein induced by keratin

In our microarray data an adhesin like protein was modulated during the growth of *T. rubrum* on keratin and also in co-culture in a keratinocyte cell. Adhesins are also required for the early stages of infection in dermatophytes [8]. The gene expression modulation of *T. rubrum* adhesin like-protein during the co-culture of conidia in keratinocytes cell line assessed by qPCR showed an increase in gene expression 6 h followed by a decrease at 12 h and a subsequent increase at 24 h. Liu et al. [16] demonstrated that dormant conidia of *T. rubrum* show a fluctuation on gene expression. During the germination process, the same work followed a different time course of conidia development and the morphological transitions promoted during the time was also evaluated indicating that at 6 h the conidia is brighter and swollen and after 12 h the hyphae begin to be developed. As the adhesins are mainly expressed in germinating conidia, as occoured for MAD1 in *M. anisopliae* [24], the result obtained by qPCR for the expression of *T. rubrum* adhesin-like protein was even expected for the incubation of 6 h. Furthermore, Aljabre et al. [25] studying the co-culture of *Trichophyton mentagrophytes* in corneocytes cells showed that the adherence of the arthroconidia requires 6 h and more than 4 h for germination. Regarding to the time of 24 h, some works also demonstrated the adherence of spores to *corneum stratum* for this time [26] . Thus, our assumption is that the adhesin-like protein has a fluctuation on gene expression according to the cell cycle process.

In *silico* analysis of this adhesin like protein showed the presence of collagen triple helix domains, mucin-like glycoprotein domain and flocullin domain, as described previously. The collagen triple helix domains are currently being investigated because of their role in host-pathogen interactions and bacterial adhesins [27]. The mucin-like glycoprotein domain of these proteins plays a role in the adhesion and pathogenicity of microorganisms, including biofilm formation [28]. The flocculin domain is present in many cell wall proteins (CWPs) with tandem repeats that are responsible for mediating the host-pathogen interaction by cell-cell adhesion, and it is related to the biofilm formation observed in *Aspergillus fumigatus* and *Saccharomyces cerevisiae* [29, 30]. The fungal adhesion process has been extensively studied in *Candida* spp. [31], but it has only begun to be addressed in other pathogenic fungi. In this respect, an adhesin called MAD1 has been characterized in the entomopathogenic fungus *Metarhizium anisopliae*. The disruption of MAD1 adhesin delays germination, suppresses blastospore formation, and reduces virulence against caterpillars [24]. Also, the cell surface protein of *Aspergillus fumigatus*, which shows features of adhesin-like protein was investigated, and a null mutant for the *cspA* gene showed a phenotype characterized by rapid conidial germination and reduced adhesion to the extracellular matrix [32]. Tandem repeats are more commonly found in cell wall proteins, and the number of repeats, as well as the length of the tandem repeat unit, can vary among different species and within isolates, promoting diversity and improving adhesion capacity [29].

### Expression of genes involved in dormancy and germination of conidia

Genes involved in the specific germination processes of conidia are interesting targets for the development of new antifungal compounds. Furthermore, the chronic infections caused by these fungi may be related to conidial dormancy because an important factor underlying chronicity is the ability of *T. rubrum* to survive as conidia inside the human body [33]. The pattern of low metabolic activity reflected by the repression of a high number of genes involved in the metabolism process, also known as quiescent status, seems to be related to conidial dormancy [16, 20]. While at the same time the induction of a few genes in energy metabolism probably are important for maintaining dormancy and initiating germination [20].

The modulation of genes encoding signal transduction system proteins and regulation of biologic process that are involved in conidial dormancy and the early stages of spore germination was also observed in the present study [34]. Moreover, we found that elastin promoted modulation of a higher percentage of genes involved in the regulation of biological processes, which may be related to the establishment of deep infections. Indeed, studies have shown that genes coding proteins required for polarized growth and WD-repeat proteins (related to the interaction of signaling molecules) appear to be important for the processes of systemic fungal infections caused by *A. fumigatus* and *C. albicans* [35, 36]. However, further studies are needed to better understand the role of signal transduction systems and regulation of biological process in the maintenance of dormancy in *T. rubrum* conidia.

### Expression of genes coding proteases

Proteases play a central role in pathogenesis, as they are widely implicated to have proteolytic activity [37, 38]. At least 20 protease gene that belong to the metalloprotease and serine protease families of proteolytic enzymes are found in the genome of *T. rubrum* and other dermatophytes [39]. Among endoproteases, there are five metalloproteases (fungalysins) and seven serine proteases (subtilisins). The exoproteases are represented by two metalloproteases, leucine aminopeptidases Lap1 and Lap2, and two serine proteases, dipeptidyl-peptidases DppIV and DppV [38]. In addition to these proteases, *T. rubrum* was also found to secrete a metallocarboxypeptidase (McpA) and to produce two membrane-anchored serine carboxypeptidases when cultured in medium containing protein as the sole nitrogen and carbon source [40]. The proteases modulated in this work, especially during the growth of *T. rubrum* on keratin, are relevant to better understand the role of these proteases for the conidia infection process.

## Conclusion

The present results broaden the knowledge of the molecular features of infection with *T. rubrum* conidia. Genes involved in conidial adhesion and dormancy seem to be important for the infection process and could be explored as potential targets for the development of new antifungal agents.

## Methods

### Strain, media and growth conditions

*T. rubrum* strain CBS118892 was cultured on Sabouraud dextrose agar (Oxoid, Hampshire, England) for 15 days at 28 °C to induce full sporulation. The conidial solution was filtered twice through glass wool to remove any hyphal fragments and was inspected by microscopy. The number of conidia was counted with a hemocytometer under a Nikon microscope. Approximately 2.6 x 10^6^ conidia/mL were added to 10 mL of three different media in triplicate: i) Cove’s minimal medium (control) containing 70 mM nitrate (Sigma Aldrich, St. Louis, MO, USA) and 50 mM glucose (Sigma Aldrich); ii) Cove’s minimal medium supplemented with 0.5 % bovine keratin; and iii) Cove’s medium supplemented with 0.25 % elastin (Sigma Aldrich). Cultures ii and iii received 3.5 mM nitrate and 2.7 mM glucose. *T. rubrum* cultures (i, ii, and iii) were incubated for 24, 36 and 72 h at 28 °C under shaking (130 rpm) and were collected by centrifugation at 1,000 *g* for 10 min.

### Co-culture conditions

The human keratinocyte cell line HaCat was grown in RPMI medium (Sigma Aldrich) supplemented with 10 % fetal bovine serum (FBS) (Sigma Aldrich) at 37 °C in 5 % CO_2_. Keratinocytes were collected, washed, and counted with a hemocytometer. A total of 2.5 x 10^5^ cells/mL were plated in 250-mL tissue culture flasks containing RPMI supplemented with 2 % FBS and grown for 24 h at 37 °C in 5 % CO_2_. The *T. rubrum* solution containing 1 x 10^7^ conidia/mL was resuspended in RPMI medium containing 2 % FBS. The solution of conidia was added to the keratinocyte cultures and incubated for 6 h, 12 h and 24 h at 37 °C in 5 % CO_2_. Fungi and human cells were collected by scraping with a rubber scraper, transferred to 1.5-mL microtubes, and centrifuged at 1,730 *g* for 10 min.

### Total RNA extraction

*T. rubrum* cultures (i, ii, and iii) grown for 24 h or co-cultured with keratinocytes were treated with lysis solution (20 mg/mL lysozyme, 0.7 M KCl and 1 M MgSO_4_, pH 6.8) for 1 h at 28 °C while shaking (130 rpm) and were collected by centrifugation at 1,000 *g* for 10 min. The cells were ground with a mortar and pestle and pulverized in liquid nitrogen. Total RNA was extracted using the Illustra RNAspin Mini RNA Isolation Kit (GE Healthcare-Little Chalfont, Buckinghamshire, UK). RNA preparations were confirmed to be free of protein and phenol by UV spectrophotometry. RNA degradation was assessed by microfluidic electrophoresis using Agilent 6000 RNA Nano chips and an Agilent 2100 Bioanalyzer (Agilent Technologies, Santa Clara, CA, USA). Only RNA samples that were free of protein and phenol and had an RNA integrity number (RIN) ≥ 9.0 were used.

### Microarray hybridization

Twenty-five nanograms of RNA from each incubation time (24, 36 and 72 h) and treatment condition (control, keratin and elastin) were pooled. Finally, 75 ng of RNA from each condition was used to synthesize double-stranded cDNA and cyanine 3 (Cy3)-CTP labelled complementary amplified RNA (cRNA) using the Agilent Low Input Amplification Kit (Agilent Technologies, Santa Clara, CA, USA) according to the manufacturer’s instructions. Agilent 4 × 44 K High-Density Oligonucleotide custom microarray slides were designed with the *e-array* tool (Agilent Technology Genomics). The ESTs (expressed sequence tags) selected were obtained from the NCBI database (www.ncbi.nlm.nih.gov/), the mapping of these ESTs with the genome of *T. rubrum* retrieved in 6,091 encoding genes. The cyanine-labelled complementary RNA was hybridized to microarrays slides (Agilent Technologies) in SureHyb chambers (Agilent) in a rotator oven for 18 h at 60 °C; two biological replicates were used for each condition. Internal control probes were included in addition to the functional genes of *T. rubrum*. The arrays were washed according to the manufacturer’s instructions and scanned with an Agilent DNA Microarray Scanner.

### Microarray data analysis

The oligo-mRNA array slides were scanned with a DNA microarray scanner (Agilent Technologies), and Agilent Feature Extraction 10.5 software was used to extract the hybridization signals. The analysis was performed by pairwise comparison of keratin x control or elastin x control. The quantitative microarray data were normalized with a 95^th^ percentile expression filter and were analysed using the Gene Spring GX 12.6 Bioinformatics Platform (http://www.agilent.com/chem/genespring)  according to the manufacturer’s instructions. Statistical analysis was performed by ANOVA (P < 0.05) using a fold change ≥ 4.0. The mapping of each EST with a protein coding gene in *T. rubrum* genome was obtained through alignments performed with Blastx (e-value 1e-5). Also, the putative annotations were retrieved using Blastx according to ncbi bank, then the biological function of putative protein was assessed through GO terms obtained with BLAST2GO 2.4.8 software. Some additional information of relevant genes was obtained from NCBI’s conserved domain database. After the Blast2GO analysis, some genes did not present any GO associated term and in these cases these genes were described as “Unclassified”. The raw data are deposited in the Gene Expression Omnibus (GEO) (www.ncbi.nlm.nih.gov/geo) database under accession number GSE 69305.

### *In silico* identification and prediction of the adhesin-like protein gene and protein sequence analysis

Annotation of the TERG_08771 gene occurred in May 2014 through the Broad Institute’s Dermatophyte Comparative Database, and the gene was assigned as a hypothetical protein. *In silico* identification of this gene was performed using the following tools: Blast2GO [41], Funcat [42], NCBI Blast, and CDD (Conserved Domain Database) [43]. Blastx from NCBI with an e-value of 1e-05 was used to identify homologies. Additionally, the TERG_08771 gene was submitted to the FaaPred prediction method for fungal adhesins and adhesin-like proteins (http://bioinfo.icgeb.res.in/faap/query.html) [9]. A tandem repeat analysis of the TERG_08771 gene between dermatophytes was performed with Tandem Repeat Finder [44] using the following parameters: matching weight, 2; mismatching penalty, 5; indel penalty, 5; match probability, 0.8; indel probability, 0.1; score ≥ 40; and maximum period, 500. Variability analysis was performed using the SERV algorithm [45].

### Quantitative RT-PCR

A set of nine genes (Table 4) was selected for quantitative RT-PCR to validate the microarray expression data. Additionally, the expression of TERG_08771 (adhesion-like protein) was evaluated in a co-culture with the keratinocyte cell line. Complementary DNA was synthesized from 500 ng of total RNA in a 20-μL reaction volume using the RevertAID H Minus First Strand cDNA Synthesis Kit (Fermentas®). The quantitative RT-PCR experiments were performed in triplicate using the SYBR Taq Ready Mix Kit (Sigma) on an Mx3300 QPCR system (Stratagene) according to Bitencourt et al. [46]. The cycling conditions were as follows: initial denaturation at 94 °C for 10 min, followed by 40 cycles at 94 °C for 2 min, 60 s at 60 °C and 1 min at 72 °C. A dissociation curve was constructed at the end of each PCR cycle to verify if a single product was amplified. Expression levels were calculated by the comparative Ct method using beta-tubulin for normalization. The reference for validation of the microarray data was Cove’s minimal medium, and for the co-culture assay the reference used was the dormant conidia solution according to Komoto et al. [47] with some modifications. The results are reported as the mean ± standard deviation of three independent experiments.

**Table 4:** Primers used in RT-PCR

Primer	Sequence	GI number	Size (bp)	Reference
Adhesin like protein	F:5′- CTGCGCAGTTGTTATCAAGAAG-3′	327302703	98	This paper
	R: 5′- GTAGGCTGGTAGTTGGGAATG-3′			
Subtilin 1	F: 5′- GCTGGCTCCAATCTACTCATAC-3′	327303325	105	This paper
	R:5′- CGCTGTATCCCTTCATCTTGT-3′			
Subtilisin 3	F: 5′- AGGTTAGTCCTGAAGCCCTCT-3′	38146042	105	This paper
	R: 5′- GCGGTCGTGCTCTACATAGT-3′			
Phosphatidyl synthase	F: 5′- CCAAGAGTCCGCCGTCTATC-3′	327309347	179	This paper
	R: 5′- GGTGTGACTTCGGCAGATGA-3′			
Glutathione synthetase	F: 5′- ACTGACTGGCTGGGAGAGAT-3′	327300656	124	This paper
	R: 5′- ACAAGCCAAGTGAGAGAGGC-3′			
MFS sugar transport	F: 5′- AAACCACCGCCTCGTTATGT-3′	327301116	127	This paper
	R: 5′- GATGGCCAAAAGACCCGGTA-3′			
Tubulin beta chain	F: 5′- AACATGATGGCTGCCACTGA-3′	10371186	253	[48]
	R: 5′ - AAGATGGCAGAGCAGGTAAGGT-3′			

### Availability of data and materials

The dataset supporting the conclusions of this article is available in the Expression Omnibus (GEO) in http://www.ncbi.nlm.nih.gov/geo database under accession number GSE 69305.
